# Is a stimulating work environment related to job satisfaction and retention among care professionals? A cross-sectional study in Dutch nursing homes

**DOI:** 10.1108/JHOM-03-2024-0085

**Published:** 2025-04-01

**Authors:** Bellis van den Berg, Yael Reijmer, Karin Kee, Henk Nies, Bianca Beersma, Mariëlle Zondervan-Zwijnenburg

**Affiliations:** Vilans, National Center of Excellence for Care and Support, Utrecht, Netherlands; Department of Organization Sciences, Vrije Universiteit Amsterdam, Amsterdam, Netherlands

**Keywords:** Job satisfaction, Job retention, Organizational conditions, Employee voice, Health care professionals

## Abstract

**Purpose:**

This study aims to examine how care professionals of different occupational groups perceive organizational conditions for voice behavior and whether these conditions are related to job satisfaction and retention as was experienced at nursing home (NH) facilities.

**Design/methodology/approach:**

An online survey study was conducted among care professionals, (middle) managers and policy officers of 175 Dutch nursing home (NH) facilities (*N* = 3,932 respondents). Data were collected between November 2020 and January 2022. Organizational conditions were clustered into four categories: dealing with incidents, formal opportunities, supportive management and teams’ improvement orientation.

**Findings:**

Certified nurse assistants (CNAs) and registered nurses (RNs) were more critical about the organizational conditions than other respondents from the same NH facility. Linear mixed effect models showed that organizational conditions were positively related to job satisfaction and perceived employee retention (*p* < 0.001). Hierarchical multiple regression models show that “teams’ improvement orientation” and “supportive management” are strong independent predictors of job satisfaction and perceived employee retention (*p* < 0.001).

**Practical implications:**

It is important that leaders realize that their own perspectives may not correspond with those of frontline care workers. This emphasizes the importance of capturing different perspectives on organizational conditions and the important role of middle managers who are in the position to create stimulating working environments to retain care professionals for NH care.

**Originality/value:**

This study has the unique opportunity to examine the relation between organizational conditions and job satisfaction among different occupational groups in Dutch NHs. Showing that care professionals were considerably more critical about the conditions and their job satisfaction compared to management and policy officers.

## Introduction

Labor market shortages and subsequent understaffing of skilled care personnel impede the quality and sustainability of elderly care around the globe. In particular, there exists a shortage of nurses, who, according to the World Health Organization (WHO, 2020) make up for more than 50% of the current global shortage in healthcare workers. To prevent this shortage from further increasing, enhancing job satisfaction, as well as nurse retention is crucial (Twigg and McCullough, 2014).

Over the last decades, nursing scholars have extensively studied conditions that influence nurses’ job satisfaction, work engagement and retention (Caricati *et al.*, 2014; Sasso *et al.*, 2019, Pinho *et al.*, 2023). This previous research has shown that having opportunities to discuss and influence one’s work conditions is an essential predictor of nurses’ job satisfaction, work engagement and retention (Bashshur and Oc, 2014; Both-Nwabuwe, 2020; Moloney *et al.*, 2020, Pinho *et al.*, 2023, Kee *et al.*, 2024). In the scientific literature, this type of communicative behavior is known as “employee voice” and is defined as “the different ways and means through which employees speak up, attempt to have a say in, and potentially influence issues that affect their work or lives” (Kee *et al.*, 2021, p. 254). Exhibiting voice behavior by openly sharing ideas and concerns regarding work-related matters or needs with those who can act upon the desired changes can actually lead to the desired organizational improvements (Morrison, 2014). Autonomy and empowerment might be psychological needs that underly this voice behavior (Kee *et al.*, 2024; Squires *et al*., 2015). Voice behavior namely means that employees take active efforts to shape their work and work environment (Morrison, 2014). Thus exercising voice could be regarded as a way for employees to experience a sense of autonomy and empowerment which in turn contributes to job satisfaction.

Given this relation between voice behavior and job satisfaction, work engagement and retention, creating work environments where care professionals feel able and willing to make their voices heard is of importance. Prior research has suggested that two conditions must be met for employees to become willing to exercise their voice (i.e. share their ideas, concerns and perspectives (Morrison, 2014). First, workers must experience a feeling of “voice safety”. They must believe that engaging in voice does not lead to personal harm or deteriorated relationships with supervisors or colleagues (Edmonson, 1999; Garon, 2012). Second, workers must experience “voice efficacy.” They must perceive that their voice will be listened to and acted upon (Morrison, 2014). Organizational conditions have been shown to affect the aforementioned employee perceptions (Kee *et al.*, 2021; Pavithra *et al.*, 2022). For instance, when leaders (i.e. team leaders, managers, directors) signal that they are open to input, this generally enhances workers’ safety and efficacy perceptions (Tangirala and Ramanujam, 2012). Hence, the organizational conditions for employee voice are strongly linked to employees’ ability and willingness to make their voices heard, which, in turn, have been shown to influence employee’s job satisfaction and retention (Garon, 2012; Edmonson, 2003; Holland *et al.*, 2011).

To date, research on voice behavior and conditions for voice behavior in healthcare has mainly been conducted among nurses working in hospitals (Moloney *et al.*, 2020; Garon, 2012; Edmonson, 2003). Since long-term care faces serious labor market problems, there is a need to better understand the relationship between organizational conditions that are associated with voice behavior, job satisfaction and retention among care professionals in nursing home care.

In Dutch nursing homes (NHs), similar to other countries, care professionals with different educational levels work together in teams. Nursing assistants (NAs) typically have two years of education, certified nurse assistants (CNAs) three years, and registered nurses (RNs) four or more years. Nursing is characterized by hierarchy caused by various educational levels with different powers and competences (Kee *et al.*, 2021; Apker and Eggly, 2004) of which RNs typically enjoy the highest status in Dutch care organizations and NAs the lowest.

The extent to which nurses are inclined to engage in voice behavior, and how they value the conditions for voice behavior, might differ between these job groups, given that prior studies have shown that those in higher job groups, who typically enjoy higher status, are more likely to speak up than those in lower job groups (Kee *et al.*, 2021; Janssen and Gao, 2015). To date, most research has been conducted among RNs. Relatively little research has been conducted among NAs and CNAs, who form the largest group of care professionals in NH in the Netherlands and who may feel less free to speak up in this hierarchical context. To be able to create the right conditions for these different groups, we need to be mindful of this diversity and different perspectives (Wilkinson *et al.*, 2018).

## Study objective

The objective of this study is to examine the following: 1) How care professionals of different job groups perceive organizational conditions for voice behavior, 2) Whether these organizational conditions for voice behavior are related to both job satisfaction and retention as was experienced at the NH facility and 3) Whether these results differ between the different job groups.

## Literature review of related studies

### Theoretical perspective of organizational conditions for voice behavior

Employee voice behavior is important because it has been related to job satisfaction and retention in prior studies (Bashshur and Oc, 2014; Both-Nwabuwe, 2020; Moloney *et al.*, 2020). Several conditions may contribute to nurses’ ability to voice in healthcare settings (Garon, 2012; Kee and De Jong, 2022). Prior research, in settings other than long-term care, has shown that social processes also affect employee voice behavior (Morrison, 2014). Such as the behavior leaders demonstrate, whether employees receive support from their peers, and the extent to which the organization has a culture of improvement and safety. In what follows, we describe four social processes and organizational conditions that have been shown to foster employee voice. In the present study, we examined whether these different social processes and organizational conditions are directly related to nurses’ job satisfaction and perceived retention.

### Dealing with incidents

The extent to which incidents are reported and framed as opportunities for teams or for organizations to learn and improve can be seen as an antecedent for voice behavior. Edmonson showed that team members’ willingness to discuss mistakes openly has primary influence on the detected error rates (Edmonson, 2004). When incidents are framed as errors, for which someone is to blame, reporting incidents can negatively affect someone’s career. This might inhibit an employee’s tendency to report and discuss incidents and therefore, inhibit employee voice behavior. On the other hand, dealing with incidents as learning opportunities may result in the implementation of improvements, which may facilitate’ voice behavior (Grant, 2013; Leistikow *et al.*, 2017; Levine *et al.*, 2020).

### Formal opportunities for employee participation

Formal opportunities for employee participation, such as participating in advisory boards or meetings with senior management, have been shown to be positively associated with job satisfaction (Holland *et al.*, 2011). Such opportunities can encourage interactions between management and employees and offer employees an opportunity to influence decisions in the organization, which have been shown to increase nurses’ job satisfaction (Both-Nwabuwe, 2020).

### Supportive management

Previous research has shown that leaders may play an important role in enhancing voice behavior. For instance, leaders can enhance workers’ voice, specifically “voice safety” and “voice efficacy”, by promoting positive and open communication and by being approachable for employees (Garon, 2012; Detert and Burris, 2007). In this regard, Nembhard and Edmonson (2006) emphasize the role of leader inclusiveness, i.e. the words and deeds exhibited by leaders that invite and appreciate others’ contributions. Tangirala and Ramanujam (2012) also showed that leaders’ consultation was positively related to nurses’ upward voice. The perception that leaders are involved, willing to listen and interested in employees’ ideas, has been shown to be beneficial to workers’ voice (Garon, 2012; Detert and Burris, 2007).

### Teams’ improvement orientation

The extent to which the team is oriented towards learning, feedback and continuous improvement may enhance voice behavior, and can therefore be seen as a precondition of voice (Edmonson, 2003). Kee and De Jong (2022), showed the extent to which colleagues encouraged and welcomed novice nurses to speak up, affected the latter’s willingness to exhibit voice. Also, in a qualitative study among RNs, participants reported that peers influenced one’s ability to speak up (Garon, 2012). When teams are oriented towards (continuous) improvement, team members may be more likely to perceive speaking up as safe and effective. Speaking out may then be viewed as part of their role, which may motivate team members to engage in voice behavior (Edmonson, 1999; Burris, 2012; Pinder and Harlos, 2001; Wilkinson *et al.*, 2004; Verbeek *et al*., 2023). Furthermore, teams’ improvement orientation is related to the concept of the “learning organization” in which employees participate in problem-solving and learning from mistakes is promoted (Watkins and Marsick, 1993). This means that team members and team leaders are open to change, they reflect and learn from situations and actions, and they are oriented towards continuous improvement, all of which has been associated with job satisfaction (Puhakka *et al.*, 2021; Rose *et al.*, 2009).

### Differences between job groups

The present study differentiates between different groups of care professionals, managers and policy officers. Since NHs are characterized by hierarchy caused by various educational levels that are accompanied with different views, powers and competences, it is important to pay attention to these different professional groups. Although, studies that compare different job groups with respect to their perceptions of organizational conditions, voice behaviors and job satisfaction are scares, differences in job satisfaction between educational levels of care professionals have been identified (Wei *et al.*, 2023; Kvist *et al*., 2013). For example, Kvist *et al*. (2013) demonstrated that physicians exhibited the highest levels of job satisfactions, while nurses and maintenance staff were the least satisfied, and office and administrative staff were fairly satisfied. Notably, a culture of participation was identified as a key factor in this regard. In NH settings, Kee *et al*. (2024) found that CNAs are less often invited to participate in formal voice opportunities compared to RNs. Furthermore, they observed that some employees lack the skills to convey their ideas, concerns and perspectives effectively. These findings underscore the importance of studying the perceptions and experiences of different job groups separately.

## Methods

### Study setting and design

Data were collected among NH facilities that participated in a nation-wide quality program that was established to improve quality of care in NHs. In the Netherlands, nearly all NH homes are publicly funded, non-profit organizations. The program was accessible to all Dutch NH facilities and they were at liberty to sign up to the program on a voluntary basis. Participation was free of charge, but NH facilities were obliged to repay back all expenses if they failed to adhere to the programme agreement and/or withdrew from the support programme without sufficient reason.

Data were collected between November 2020 and January 2022, at the start of participation in the quality improvement program in 175 residential NH facilities. These NH facilities belonged to 61 public NH home organizations with a number of facilities ranging from one to thirty. The participating NH organizations were characterized by on average 170 residents per organization, with on average 200 care professionals per organization, including (para)medical, auxiliary and psychosocial staff. In the Netherlands, care professionals in NH are generally well qualified. A slight majority of the participating NH organizations were located in the western urban areas of the Netherlands, with the remainder situated in more rural locations. The western urban areas comprise the provinces of North–Holland, South-Holland, Flevoland and Utrecht. These areas are characterized by the presence of several agglomerations of large cities, a concentration of industry and a high population density. In these areas, which are together also known as “the Randstad”, the shortage of skilled NH personnel is greater than in rural areas, while the number of older people requiring NH care is increasing at a similar rate.

### Data collection and study population

In the improvement programme, a comprehensive problem analysis per facility was conducted using a revised version of the Quality Evaluation Questionnaire for Nursing Homes (QEQ-NHs) (Triemstra *et al.*, 2021). This instrument was developed in 2019 by Vilans (National Center of Expertise for Long-Term Care and Support in the Netherlands). The revised version of the questionnaire was developed through the use of cognitive interviews with the various respondent groups (Buers *et al.*, 2014). Psychometric analyses were subsequently conducted to confirm the reliability of the revised questionnaire. The QEQ-NH revised comprises a number of questionnaires designed to assess the (conditions for) quality of care in NH from different perspectives, including those of residents, family, care professionals, management and policy officers.

The instrument encompasses the themes described in the Dutch National Quality Framework for Nursing Home care including items pertaining conditions that are associated with voice behavior, job satisfaction and perceived staff retention at the facility. Questions are formulated as propositions, and answering scales range from 1 (completely disagree) to 5 (completely agree). All propositions were formulated such that a higher score indicates a more favorable situation. Such Likert values are technically ordinal, as equidistance between the scores cannot be guaranteed. Nevertheless, they are commonly considered robust for calculating averages and performing statistical analyses (Sullivan and Artino, 2013).

At the start of the improvement trajectory, the questionnaires were distributed among the different groups involved within the NH facility during a four-week period. The online questionnaire was distributed via a generic link (URL). The facility manager sent this link to all those involved within the NH facility, along with information about the quality improvement program and the importance of completing the questionnaire. By completing the questionnaire, respondents were given the opportunity to share their experiences with the (conditions for) quality at the facility. All residents, family, care professionals, policy officers and management were asked to fill out the questionnaire, but participation was voluntary. Facilities were encouraged to have at least 20 to 50% of their employees and family members complete the questionnaire. If too few questionnaires were completed, a reminder was sent by the facility manager.

In the present study, only questionnaires filled in by nurses (NAs, CNAs and RNs), (middle) management and policy officers are included. In total, *N* = 3,932 employees of 175 NH facilities completed the questionnaire. The median number of respondents per facility was 20 (range 4–65). Table 1 displays the number of respondents per job group.

**Table 1:** Number of respondents per job group

	Total number of facilities	Total number of respondents
Nurse assistant (NA)	160	859
Certified nurse assistant (CNA)	175	2,026
Registered nurse (RN)	161	573
Middle management	96	158
Management/policy officer (reference)	136	316
Max/total	175^a^	3,932

**Note(s):** ^a^Data from all three caregiver groups (NA, CNA and RN) were available from 149 residential facilities

**Source(s):** Authors’ work

### Data analysis

#### Independent variables

Based on the literature, 10 items from the QEQ-NH that relate to organizational conditions for voice behavior were selected for this study and clustered into four categories: 1) *Dealing with incidents* (2 items), 2) *Formal opportunities for employee participation* (1 item), 3) *Supportive management* (2 items) and 4) *Team’s improvement orientation* (four items). Appendix shows the different questionnaire items that correspond with the four categories, the questionnaire items were adapted to each group according to their role.

#### Reliability

Although the four constructs of organizational conditions were based on the literature, we also tested the reliability of the composed constructs. Cronbach’s alpha was calculated for the construct Team’s improvement orientation (four items), showing good internal consistency (α > 0.81). Since Cronbach’s alpha is often underestimated for scales with only 2 items, the Spearman-Brown inter-item correlation was used as a measure of reliability (Eisinga *et al.*, 2013) for the construct of “Dealing with incidents”, with a minimum threshold of 0.20 (Pallant, 2011). All inter-item correlations were >0.40. Table 2 shows the number of items, the inter-item correlation and Cronbach’s alpha for the construct Team’s improvement orientation.

**Table 2:** Number of items, inter-item correlation and Cronbach’s alpha (α) of three constructs of perceived preconditions for voice behavior

Preconditions for voice behavior^a^	No. of items	Inter-item correlation	Cronbach’s α
Dealing with incidents	2	0.56	
Supportive management	2	0.88	
Teams’ improvement orientation	4		0.81

**Note(s):** ^a^Constructs were formed based on theory. Inter-item correlations are given for the 2-item construct

**Source(s):** Authors’ work

#### Outcome variables

We studied two different outcomes: job satisfaction and retention as was experienced by employees at the NH facility. Job satisfaction was defined as the feeling of enjoyment that employees derived from their job and was measured by one single proposition that was formulated as: “*I enjoy going to work”.* Respondents were asked to indicate their level of agreement on a five-point Likert scale, with 1 representing the most negative response and 5 the most positive. Perceived retention was a composite indicator for employee retention as was experienced at the facility based on two items: 1) the average of the experienced sick leave at the NH facility (*“Employees are rarely absent due to sick leave, this is not a structural problem”*) and 2) the average of the experienced staff turnover (“*There are few changes in the teams and the turnover of staff is not a structural problem”).* For these items, respondents were also asked to indicate their level of agreement on a five-point scale, with 1 representing the most negative response and 5 the most positive. As the respondents were asked to evaluate the employee retention based on their experience at the facility, the data on perceived employee retention (employee turnover and sickness absence) were averaged to obtain one single measure for each NH facility (Cronbach’s alpha: 0.80). We used full information maximum likelihood estimation (FIML, Graham, 2009) to handle missing values on these outcome measures.

All analyses were performed with Mplus version 8.7 (Muthén and Muthén, 2012). As the participants in this study are nested in facilities, which, in turn, are nested in organizations, we conducted hierarchical analyses to model this nonindependence and correct for underestimated standard errors.

Differences among job groups (5 levels) in perceived organizational conditions for voice behavior and differences in job satisfaction at the individual level were evaluated in a hierarchical model. We tested the hypothesis that all group means are equal with a Wald test. The Wald test is a test for parameter constraints. Post-hoc evaluations were conducted with management and policy officers (i.e. the group with the highest authority) as the reference group.

Next, we examined the (simple) relations between conditions for voice behavior (independent variable) and job satisfaction (dependent variable) for the three caregiver groups (NA, CNA and RN) in hierarchical multigroup regression models. In these models, we evaluated differences in regression coefficients between caregiver groups as well.

Additionally, we examined the (simple) relations between conditions for voice (independent) and the degree of retention as was experienced at the facility with hierarchical linear regression models. As respondents were to evaluate employee retention as they experienced it at the facility level, we calculated a mean score for the perceived employee retention at each facility. Therefore, we did not evaluate associations for separate caregiver groups.

Finally, we examined which of the four organizational conditions for voice were independently related to job satisfaction and employee retention at the facility with hierarchical multiple regression models. For the outcome variable job satisfaction, we stratisfied by job group.

#### Ethical considerations

All data were collected and handled in accordance with the relevant privacy protection guidelines. According to Dutch law, our study does not fall under the remit of the “Medical Research Involving Human Subjects Act”, and no approval by a medical ethics committee was necessary. This act is based on the Helsinki declaration of the World Medical Association and is executed by the Central Committee on Research Involving Human Subjects (CCMOs). Verbal consent was obtained from respondents who completed a questionnaire anonymously, and their response expressed their willingness to participate.

E-mail addresses and contact information for employees, volunteers and family members were not collected by the program, because of privacy reasons and because this information was not necessary for the purpose of the improvement program. In addition, because the data were collected primarily to gain insight into the quality and conditions for quality of care at the facility, we did not collect information about the demographic background of the participants. This was done to ensure the anonymity and privacy of the participants. Furthermore, the outcomes were discussed in the facilities for improvement reasons. In order to ensure open discussions without any blaming or shaming, anonymity was an essential requirement. Socio-demographic details might have challenged this anonymity as the communities within some of the participating facility were quite small.

## Results

### Organizational conditions and job satisfaction as perceived by care professionals of different job groups

**Figure 1:**
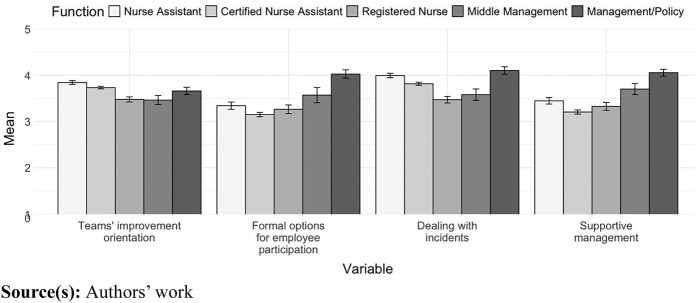
Mean score on organizational conditions for voice behavior per job group

Figure 1 shows the mean score of the organizational conditions by job group. The U-shape curve of this figure indicates that respondents from the middle job groups (CNA and RN) are more critical about the conditions for voice behavior than other respondents from the same NH facility (overall effect of job group *p* < 0.001). Post-hoc analysis showed that NAs, CNAs, RNs and middle management assigned lower scores than management and policy officers (reference group) to the conditions “formal voice” and “management support”. CNA, RN and middle management assigned lower scores to the condition “dealing with incidents” than the reference group. NA and RN also assigned lower scores than management and policy officers to the condition ‘“teams” improvement orientation’ (*p* < 0.01; Table 3). In other words, managers and policy officers had a rosier picture of the four conditions for voice behavior compared to the other respondent groups.

**Table 3:** Effect of job group on perceived preconditions for voice behavior

	Overall effect of job group^a^	Post-hoc test result: lower score than management/policy *p* < 0.01
Teams’ improvement orientation	106(4), *p* < 0.001	NA, RN, MM
Formal opportunities for employee participation	223(4), *p* < 0.001	NA, CNA, RN, MM
Dealing with incidents	197(4), *p* < 0.001	CNA, RN, MM
Supportive management	180(4), *p* < 0.001	NA, CNA, RN, MM

**Note(s):** ^a^Results hierarchical analysis with omnibus Wald test. Data show *χ*^2^(df), *p*-value. “Managers and policy officers” was used as the reference group in post-hoc analysis. NA = nurse assistant; CNA = certified nurse assistant; RN = certified nurse, MM = middle management

**Source(s):** Authors’ work

**Figure 2:**
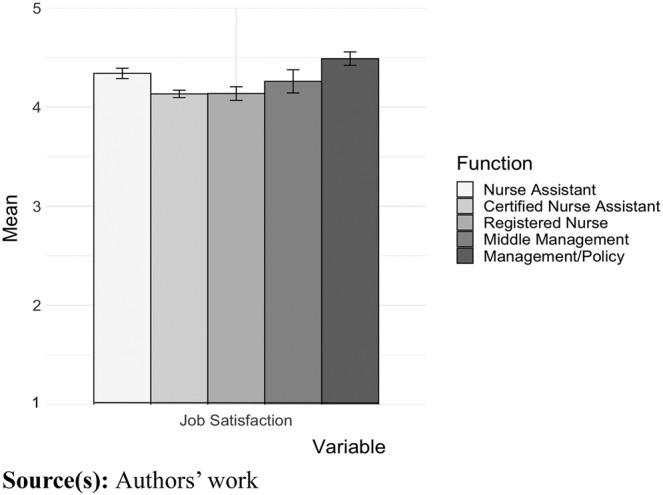
Mean score on job satisfaction per job group

Figure 2 shows the mean score of the outcome variable, job satisfaction. This figure also shows a U-shape curve, with the lowest scores among the CNA and RN groups. Post-hoc analysis showed that NAs, CNAs RNs and middle managers reported lower job satisfaction than the managers and policy officers did (*p* < 0.01). The mean score for retention at the *N* = 175 NH facilities was 2.71 (range 1.20–4.67).

### Univariate relationships between organizational conditions, job satisfaction and retention

Univariate linear mixed effect models showed significant associations between all perceived conditions for voice behavior and job satisfaction for the three caregiver groups (Table 4) and between the conditions and perceived employee retention at the NH facility (Table 5). These results showed that perceived conditions for voice behavior are positively related to both job satisfaction and retention.

**Table 4:** Association between organizational preconditions and employee job satisfaction for different job groups^a^

	Job satisfaction
*Team’s improvement orientation*	
Nurse assistants (NA)	0.445 ± 0.035***
Certified nurse assistants (CNA)	0.415 ± 0.023***
Registered nurses (RN)	0.311 ± 0.050***
*Formal opportunities for employee participation*	
Nurse assistants (NA)	0.308 ± 0.049***
Certified nurse assistants (CNA)	0.320 ± 0.029***
Registered nurses (RN)	0.358 ± 0.043***
*Dealing with incidents*	
Nurse assistants (NA)	0.353 ± 0.039***
Certified nurse assistants (CNA)	0.307 ± 0.025***
Registered nurses (RN)	0.190 ± 0.043***
*Supportive management*	
Nurse assistants (NA)	0.442 ± 0.040***
Certified nurse assistants (CNA)	0.385 ± 0.027***
Registered nurses (RN)	0.452 ± 0.041***

**Note(s):** Standardized regression estimates + - standard error are given. *p* = *< 0.05; **<0.01; ***<0.001. ^a^Job satisfaction was measured by the statement: “*I enjoy going to work”.* Respondents were asked to indicate their level of agreement on a five-point Likert scale, with 1 representing the most negative response and 5 the most positive

**Source(s):** Authors’ work

**Table 5:** Association between organizational preconditions and employee retention as was experienced at the NH facility^a^

	Perceived employee retention at the facility
Team’s improvement orientation	0.606 ± 0.047***
Formal opportunities for employee participation	0.467 ± 0.077***
Dealing with incidents	0.490 ± 0.057***
Supportive management	0.580 ± 0.051***

**Note(s):** Standardized regression estimates + - standard error are given. *p* = *< 0.05; **<0.01; ***<0.001. ^a^Retention was based the average two items: *“Employees are rarely absent due to sick leave, this is not a structural problem”* and “*There are few changes in the teams and the turnover of staff is not a structural problem”.* For these items, respondents were also asked to indicate their level of agreement on a five-point scale, with 1 representing the most negative response and 5 the most positive. The data on these items were averaged to obtain one single measure for each NH facility

**Source(s):** Authors’ work

To statistically test whether the associations in Tables 4 and 5 were significantly stronger for a specific job group, we added a job group*organizational condition interaction term to the models. These models show weaker relationships for RNs as compared to (certified) nurse assistants in two instances: the relationship between 1) team’s improvement orientation and job satisfaction and 2) dealing with incidents and job satisfaction. The other relationships do not significantly differ between job groups.

### Organizational conditions as independent predictors for job satisfaction among care professionals and retention at the facility level

Finally, we examined whether the four perceived conditions for employee voice were also *independently* related to job satisfaction for each job group and for employee retention as experienced at the facility by multiple regression analyses. All four conditions for voice behavior were simultaneously added as predictors to the model. Tables 6 and 7 show that the conditions “Team’s improvement orientation” and “supportive management” were strong independent predictors for job satisfaction across all three job grades and for employee retention as experienced at the NH facility. This means that these two conditions explain variation in job satisfaction and retention over and above the other two conditions (“formal opportunities for employee participation” and “dealing with incidents”). In addition, “Formal opportunities for employee participation” was an independent predictor for job satisfaction among RNs, and the construct “Dealing with incidents” was an independent predictor for job satisfaction for CNA group.

**Table 6:** Multiple regression results showing associations between organizational preconditions and job satisfaction for different job groups

	Nurse assistant	Certified nurse assistant	Registered nurse
Team’s improvement orientation	0.269 ± 0.057***	0.277 ± 0.033***	0.173 ± 0.059**
Formal opportunities for employee participation	−0.028 ± 0.062	0.051 ± 0.034	0.178 ± 0.043***
Dealing with incidents	0.117 ± 0.054	0.082 ± 0.031**	−0.022 ± 0.052
Supportive management	0.295 ± 0.061***	0.191 ± 0.040***	0.312 ± 0.054***

**Note(s):** Estimate ± SE; *p* = **<0.01; ***<0.001

**Source(s):** Authors’ work

**Table 7:** Multiple regression results showing associations between organizational preconditions and perceived employee retention at the nursing home facility^a^

	Total group of care professionals
Team’s improvement orientation	0.348 ± 0.094***
Formal opportunities for employee participation	0.056 ± 0.079
Dealing with incidents	0.053 ± 0.097
Supportive management	0.282 ± 0.077***

**Note(s):** Estimate ± SE; *p* = *<0.05; **<0.01; ***<0.001. ^a^Retention was based the average two items: *“Employees are rarely absent due to sick leave, this is not a structural problem”* and “*There are few changes in the teams and the turnover of staff is not a structural problem”.* For these items, respondents were also asked to indicate their level of agreement on a five-point scale, with 1 representing the most negative response and 5 the most positive. The data on these items were averaged to obtain one single measure for each NH facility

**Source(s):** Authors’ work

## Discussion

In light of the growing nursing shortages, it is important to ensure that care professionals stay satisfied with their jobs and are willing to remain working in their current positions. Previous research has shown that having opportunities to discuss and influence one’s work conditions, also known as voice behavior, is an essential predictor of nurses’ job satisfaction, work engagement and retention (Bashshur and Oc, 2014; Both-Nwabuwe, 2020; Moloney *et al.*, 2020, Kee *et al.*, 2021, Pinho *et al.*, 2023, Kee *et al.*, 2024). Given this relation between voice behavior and both job satisfaction and employee retention, creating supporting work environments where care professionals are able and willing to exhibit voice behavior is of importance. In this study, we examined how care professionals of different job groups perceive organizational conditions for voice behavior and whether these organizational conditions for voice behavior are related to their job satisfaction and the degree retention as was experienced at the NH facility. We also examined whether these results differ between the NAs, CNAs and RNs. The findings underscore the importance distinguishing between groups of care professionals, as they may attach disparate values to specific conditions. Furthermore, our findings demonstrate that creating a supporting environment is important for maintaining job satisfaction and retention among care professionals in NHs.

First, our results show that care professionals, especially CNAs and RNs are significantly more critical about organizational conditions that are associated with voice behavior than managers and policy officers (reference group). These latter seemed to be significantly more positive about the presence of these conditions. Also, our results show that all three groups NA, CNA, RN as well as middle managers reported lower job satisfaction than managers and policy officers did (*p* < 0.01). This finding could possibly be partly explained by the fact that the data were collected during the COVID-19 epidemic. Care professionals were experiencing major consequences of this epidemic (i.e. due to increased risk of being infected, and different preventive measures), while this may have had less influence on the work experience of managers and policy officers. Despite this, our findings were in line with previous studies reporting higher job satisfaction among managers compared to nurses (Kvist *et al.*, 2013).

Second, our results show that the teams’ improvement orientations and supportive management were significant and independent predictors for job satisfaction among all three job groups. Whereas formal opportunities for employee participation was a significant and independent predictor for job satisfaction among RNs, but not for NA and CNAs. In the study by Kee *et al*. (2024), among Dutch CNAs and RNs, it appeared that CNAs were less often invited to participate in formal voice opportunities compared to RNs. In the present study, CNAs seem to value these formal opportunities less than RNs. For CNAs, dealing with incidents (the extent to which care professionals are encouraged to report and learn from incidents) appeared to be an independent and significant predictor for job satisfaction but not for NAs and RNAs. Although studies that differentiate between job groups of care professionals are scarce, systematic reviews of care professionals in long-term care and RNs in hospital settings have also found different factors to be important for care professionals with different roles. For example, in these reviews both RNs’ job satisfaction and (C)NAs’ job satisfaction appeared to be closely related to working conditions, but (C)NAs noted workloads and availability of facility level resources as important while registered hospital nurses noted team cohesiveness and physical conditions of the unit to be important (Squires *et al.*, 2015; Lu *et al.*, 2012).

Third, multivariate models showed that “supportive management” was an independent predictor for job satisfaction among all three groups of care professionals as well as for retention as was experienced at the facility level. This was is in line with previous studies that showed leadership and support from management and supportive work environment are major factors that impact job satisfaction among care professionals (Lei *et al.*, 2022; Levine *et al.*, 2020; Morrow *et al.*, 2016). In particular, in a study by Chamberlain *et al.* (2016) among NAs in long-term care supervisor leadership was a significant factor contributing to job satisfaction of these care professionals.

## Conclusion

In the light of the major shortages of NAs, CNAs and RNs in long-term care, the lower job satisfaction and their more negative perceptions of conditions for voice behavior compared to management and policy officers is a point of attention. This is especially relevant given the strong relationship between organizational conditions, particularly the presence of supportive management, and job satisfaction among these care professionals as well as the degree of employee retention at the facility.

This study also emphasizes the importance of differentiating between groups of care professionals which each might have their own needs and factors that impact their job satisfaction.

## Practical implications

The results of this study show that it is important to pay attention to different factors and needs that contribute to job satisfaction of different groups of care professionals. Interventions to enhance job satisfaction should therefore be tailored to these different groups. Also care professionals were more critical about organizational conditions then management and policy officers. Since managers and policy officers have both the ability and the responsibility of creating supporting conditions for voice behavior, it is important that they realize that their perspectives may not correspond with those on the frontline of the organization.

The findings suggest that interventions focusing a positive team culture, enhancing collaboration and creating a supportive environment may be more important than arranging formal opportunities for participation and voice behavior. It is possible, however, that the latter will receive greater attention from management and policy officers that are not involved the frontline of the organization. In the Netherlands, emphasis is placed on formal opportunities for participation. In fact, from 1 July 2023 onwards, participation of care professionals is legally regulated by law. While such an initiative is important, as nurses are usually not represented in consultative and representative bodies in their organization (Kee *et al.*, 2021), our multivariate analyses showed that the construct of “formal opportunities for employee participation” was an independent predictor for job satisfaction only for RNs. This might be explained by the fact that in practice, only a few employees can participate in formal bodies representing these groups. Also, perhaps participation is most obvious for RNs, managers may unintentionally forget to invite CNAs to participate in these committees and councils” (Kee *et al.*, 2021). On the other hand, a supportive management was found to be an important independent predictor for both job satisfaction and perceived retention for all job groups. Also, for NAs and CNAs, the teams’ improvement orientation was also a significant predictor of their job satisfaction. So, in addition to ensuring that care professionals can have a formal say in their organization, our results show that it is just as important for leaders and nurses create a culture of learning and improvement, to get to know one another, build relationships and build a supportive environment in which it is safe to speak up (Kee *et al.*, 2021; Kee and De Jong, 2022).

The rosier picture of managers and policy officers underscores the value of capturing different perspectives on organizational conditions and ensuring that the voices of care professionals are heard. This may help to identify blind spots in the organization that may otherwise result in dissatisfaction or an undesirable turnover of staff. This highlights the important role of middle managers. Middle managers are in the position to involve care professionals, create stimulating environments with open communication within teams and to develop teams’ improvement orientation and related practices (Verbeek *et al.*, 2023). In addition, middle managers, who are positioned between work floor and higher management, might know both sides of the story and might therefore be possible to bridge the gap between employees and higher management.

## Limitations and future research

In this study, we were in the unique position to analyze data from 3,932 employees from 175 NH facilities in the Netherlands. The dataset allowed us to analyze and compare the experiences of care professionals of different job groups, managerial groups and staff within this study. The data show that CNAs and RNs are more critical about organizational conditions for voice and NAs, management and policy officers are less critical. This study contributes to the existing literature by showing strong relationships between perceived conditions for voice behavior and job satisfaction and employee retention as was experienced at the NH facilities.

This study has some limitations. First, the data that were used in this study were collected as part of a large scale quality improvement program. While this provided us the opportunity to study the perceptions of a substantial number of employees across diverse job groups, this approach does have certain limitations with regard sample size determination and conducting non-response analysis. All employees in participating NH facilities were able to complete the questionnaire, and facilities were encouraged to achieve a minimal response rate of 20%. The actual response rates varied between facilities, the median number of respondents per facility was 20 (range 4–65). Despite this, we believe that it is unlikely that the observed relations are affected by bias due to selective response, since both satisfied and unsatisfied employees participated as well as NH facilities with favorable and unfavorable organizational conditions.

Second, our dataset does not contain data of actual voice behavior and actual retention in the NH facilities. It would be interesting to study how perceived conditions for voice are related to job satisfaction and retention via their relationships with actual voice behavior, or whether the organizational conditions we examined have a positive effect by themselves and actual voice does not need to occur for this effect to materialize. Our quantitative dataset does not allow us to study the underlying mechanisms relating the conditions for voice to job satisfaction and perceived retention. Although this is a limitation, there are a number of studies showing that the perceived conditions are related to actual voice, and in this study, we relate these conditions to job satisfaction and perceived retention of care professionals (Morrison, 2014; Edmonson, 1999; Garon, 2012; Kee *et al.*, 2021).

Future research should further investigate the mechanisms underlying the relationship between the organizational conditions of voice and job satisfaction and retention among care professionals in long-term care. Research focusing on teams’ improvement orientation and supportive management might provide valuable insights and further directions on how to retain care professionals for long-term care.

In our study, NAs (the lowest-status group) seemed to be less critical about the presence of conditions for voice behavior. These results might indicate that organizational conditions for voice are less important to the NA group, compared to the CNA and RN group. We know from practice that NAs often have a hands-on mentality which might suggest that NAs might feel less need to engage in voice and might therefore be less critical about the conditions for voice. In addition, they may not see it as their job or their responsibility to voice ideas or concerns (Tangirala *et al.*, 2013). Our data do not allow us to test these hypotheses or to explain this finding. Examining personal and organizational motivators and inhibitors for voice behavior among this low-status group of caregivers is an interesting direction for future research.

Also, the Dutch NH facilities that were included in our study are publicly funded, non-profit organizations. In many other countries, NHs are for-profit organizations. This ownership has previously been linked to job satisfaction and staff turnover (Lindmark *et al.*, 2023; Heponiemi *et al*., 2011). For example, in a study by Choi *et al*. (2012), a supportive environment was related to job satisfaction in all ownership types, but nurses working in for-profit NHs were less satisfied than nurses working in non-profit organizations. This means that the organizational ownership is a factor to take into account in future studies examining job satisfaction and retention in NHs.

Furthermore, managers at the participating NH facilities demonstrated a more favorable perception of the organizational conditions that facilitate voice behavior, in comparison to the care professionals. Future studies could further investigate these different perspectives on organizational conditions and may identify strategies to bridge the gap between the experiences of care professionals and those (higher) management and policy officers. Finally, we have highlighted the important role of managers in fostering stimulating working environments. Future research could focus on the insights, methods and behaviors of middle managers in the creation of stimulating environments that retain care professionals in NH care.

## Appendix

**Table A1:** Questionnaire items corresponding with constructs of perceived organizational preconditions

Construct	Subject	Item in questionnaire for care professionals and middle management	Item in questionnaire for management and policy officers
Dealing with incidents	Reporting incidents	Care professionals do not experience any barriers to report incidents and adverse events: it is easy and it is encouraged by each other and by managers	We have clear agreements with employees about preventing and dealing with incidents and errors
	Following up incidents	In case of incidents of adverse events, the team invents and implements improvements. The resident and/or relatives are kept informed	We have clear agreements with employees about the follow-up of incidents and errors
Formal opportunities for employee participation	Nursing advisory board	Care professionals can influence the policy of the organization through the nurse/nursing advisory board	We use the advice that comes from the various advisory councils such as the nurse/nursing advisory board
Team’s improvement orientation	Collaboration in teams	Care professionals work well together. They inform each other about what has happened or what has been agreed	NA
	Reflection and discussion	Employees think critically about how their work can be improved and discuss this with each other	We encourage an open culture in which employees reflect on their work and discuss with each other what could be improved
	Evaluation of practices	Care professionals regularly evaluate working methods and procedures and adjust them when necessary	The working methods and procedures are regularly evaluated and adapted if necessary
	Culture of learning and improvement	Care professionals think critically about how their work can be improved and discuss this with each other and provide feedback to residents, relatives and each other about what has been done with it	There are clear agreements with regard to learning and improving the quality of care and we support employees so that they can act accordingly
Supportive management	Management involvement	The board and management is involved, they know what is going on at the facility	The board and management is involved, they know what is going on at the facility
	Management support	The board and management know what care professionals need to be able to perform their work properly and support them in this	The board and management know what care professionals need to be able to perform their work properly and support them in this

**Source(s):** Authors’ work
